# A novel genotyping system based on site polymorphism on spike gene reveals the evolutionary pathway of porcine epidemic diarrhea virus

**DOI:** 10.1002/imo2.70013

**Published:** 2025-04-06

**Authors:** Mingkai Lei, Huimin Li, Xiaoyu Chen, Xiaozhen Li, Xuexiang Yu, Shengnan Ruan, Hao Wu, Ahmed H. Ghonaim, Ziyang Yan, Wentao Li, Qigai He

**Affiliations:** ^1^ National Key Laboratory of Agricultural Microbiology, College of Veterinary Medicine Huazhong Agricultural University Wuhan China; ^2^ Yunnan Southwest Agricultural and Animal Husbandry Group Co, Ltd Kunming China; ^3^ Desert Research Center Cairo Egypt; ^4^ Hubei Hongshan Laboratory Wuhan China

**Keywords:** adaptive selection, evolutionary pathway, genetic polymorphism, genotyping, intraspecific recombination, porcine epidemic diarrhea virus

## Abstract

Porcine epidemic diarrhea virus (PEDV) is a lethal coronavirus in neonatal piglets characterized by rapid evolution in both genotype and phenotype. However, the underlying genetic mechanism has not been completely elucidated. In this study, we investigated and characterized PEDV field strains circulating in China between 2021 and 2022, which revealed significant genetic divergence. To improve the classification of PEDV, we developed a site‐polymorphism‐based genotyping system utilizing global PEDV genetic sequences from public databases. While there are currently multiple genotypic classification‐based systems for PEDV, our proposed approach could offer more stable classification by considering both genetic characteristics and evolutionary dynamics. Our analysis indicates that the most prevalent PEDV lineages originated from South Korea and China, with G2c: L4 predominating in China and G2c: L10 in the United States. Importantly, discrete phylogenetic analysis revealed potential evolutionary pathways of PEDV, showing that the termini of the S gene are prone to recombination, while adaptive selection is evident in the middle region. Overall, our findings provide a complementary and practical methodology for PEDV classification and offer novel insights into the evolutionary pathway of coronaviruses.

## Highlights

- This study reconstructed the global epidemic dynamics of porcine epidemic diarrhea virus (PEDV) through comprehensive big‐data analytics and proposed a novel typing system based on site‐specific genetic polymorphism.
- We first characterized the unique recombination pattern in the S gene of PEDV, highlighting the critical role of genetic recombination in driving genotypic divergence of this virus.
- Our research provides a fundamental theoretical framework for understanding the evolutionary mechanisms of PEDV and related coronaviruses. It also supports the development of prevention and control strategies.

## Graphical abstract

This study focuses on porcine epidemic diarrhea virus (PEDV), a highly virulent and rapidly evolving coronavirus threatening global swine production. We established a novel genotype system of PEDV targeting site‐specific polymorphisms in the spike (S) protein. The system demonstrates that frequent recombination events at both termini of the S gene serve as a critical mechanism driving genotypic diversity in PEDV. Together with adaptive evolution, these processes collectively shape the evolutionary dynamics of global PEDV strains. The research provides critical theoretical foundations for understanding PEDV genome evolution patterns and developing prevention and control measures.

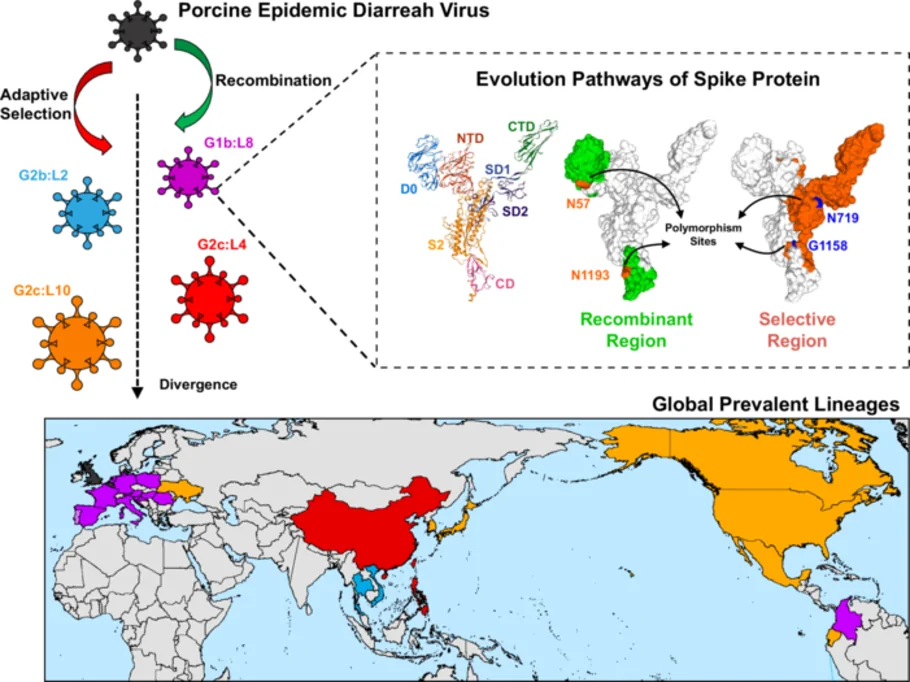

## 1 INTRODUCTION

The viral evolution and the host immune response engage in a dynamic “arms race” [1, 2], especially regarding the evolution of transmissibility and immune evasion capabilities of severe acute respiratory syndrome coronavirus‐2 (SARS‐CoV‐2) and influenza virus, which may pose significant challenges to public health and vaccine development [3, 4, 5]. Virus evolutionary biology integrates the data of viral genetic sequences, spatial‐temporal distributions, and host to elucidate the mechanisms of viral evolution [6, 7]. Specifically, the application of advanced bioinformatics methodologies can facilitate large‐scale viral genomics analysis, including phylogenetic tree construction, genotype classification, epitope prediction, and recombination events detection [8].

Porcine epidemic diarrhea (PED) is a swine disease that is characterized by severe vomiting, watery diarrhea, and dehydration, resulting in a high mortality rate among infected suckling piglets [9]. Since it was first reported in the UK in the 1970s, PED has been widely spread in Europe, Asia, and the Americas, leading to significant economic losses in the pig industry [10]. Vaccination remains the primary strategy for preventing and controlling PED in pig farms. However, due to the continuous mutation of the virus, instances of vaccination failure have become increasingly common. For example, the emergence of variant strains in 2010 in China demonstrated significant changes in both pathogenicity and antigenicity [9, 11]. These variant strains could break through the herd immunity established by the CV777‐based inactivated vaccine, leading to large‐scale outbreaks with high rates of illness and death among suckling piglets [11, 12]. Although commercially available attenuated vaccines derived from variants strains have been approved in China [13], recent outbreaks hint that these vaccines may not provide full protection against prevailing strains [14]. Furthermore, a novel variant characterized by distinct genetic traits and mild pathogenicity has been reported in both the United States and China [15, 16], thereby adding complexity to the strain diversity of porcine epidemic diarrhea virus (PEDV).

Like many other mammalian coronaviruses, PEDV belongs to the genus *Alphacoronavirus*. The genome is a 28k nt single‐stranded RNA, encoding a polyprotein, ORF1, and four structural proteins: spike (S), envelope (E), membrane (M), and nucleocapsid (N), as well as an accessory protein, ORF3 [17]. The S protein is a type I glycoprotein that can be cleaved by proteases into two subunits: S1 and S2. The S1 subunit plays a crucial role in the recognition of host cell receptors, while the S2 subunit is responsible for subsequent viral entry [18]. As observed on SARS‐CoV‐2 and human coronavirus NL63 (HCoV‐NL63) [19, 20], the extensive N‐glycosylation on the surface of S protein could modulate its biological functions and facilitate viral immune evasion. Given that variations in the S gene are linked to viral pathogenicity and contribute significantly to the overall genetic diversity of PEDV [21], the S gene is commonly utilized as an important molecular marker for investigating viral genetic evolution and genotyping [16, 22].

Recombination represents an important evolutionary pathway of coronaviruses, in which interspecific recombination may lead to host switching and the emergence of novel diseases. Notable examples include severe acute respiratory syndrome coronavirus‐1 (SARS‐CoV‐1) and Middle East respiratory syndrome coronavirus (MERS‐CoV), which have posed significant threats to public health, as well as two emerging porcine enteric coronaviruses, porcine deltacoronavirus (PDCoV) and swine acute diarrhea syndrome coronavirus (SADS‐CoV) [23, 24, 25, 26, 27]. However, interspecific recombination requires the co‐occurrence, co‐localization of two different viruses, which is typically considered a low‐probability event in nature [27]. In contrast, intraspecific recombination among different strains of the virus is more common within a multi‐strain RNA virus ecological system, potentially leading to antigenic shift and the emergence of novel phenotypes [28]. PEDV is hypothesized to originate from cross‐species transmission of bat coronavirus [29]. Additionally, frequent intraspecific recombination among various PEDV strains has been reported [22, 30].

To better describe the evolutionary biology of PEDV, we employed a variety of bioinformatic approaches and extensive genetic datasets to reconstruct the epidemic dynamics of PEDV. Our results also indicate that, in addition to adaptive selection, recombination of the S gene may have played a significant role in shaping the genogroups of PEDV.

## 2 RESULTS

### Molecular characteristics of prevalent PEDV in China

**FIGURE 1:**
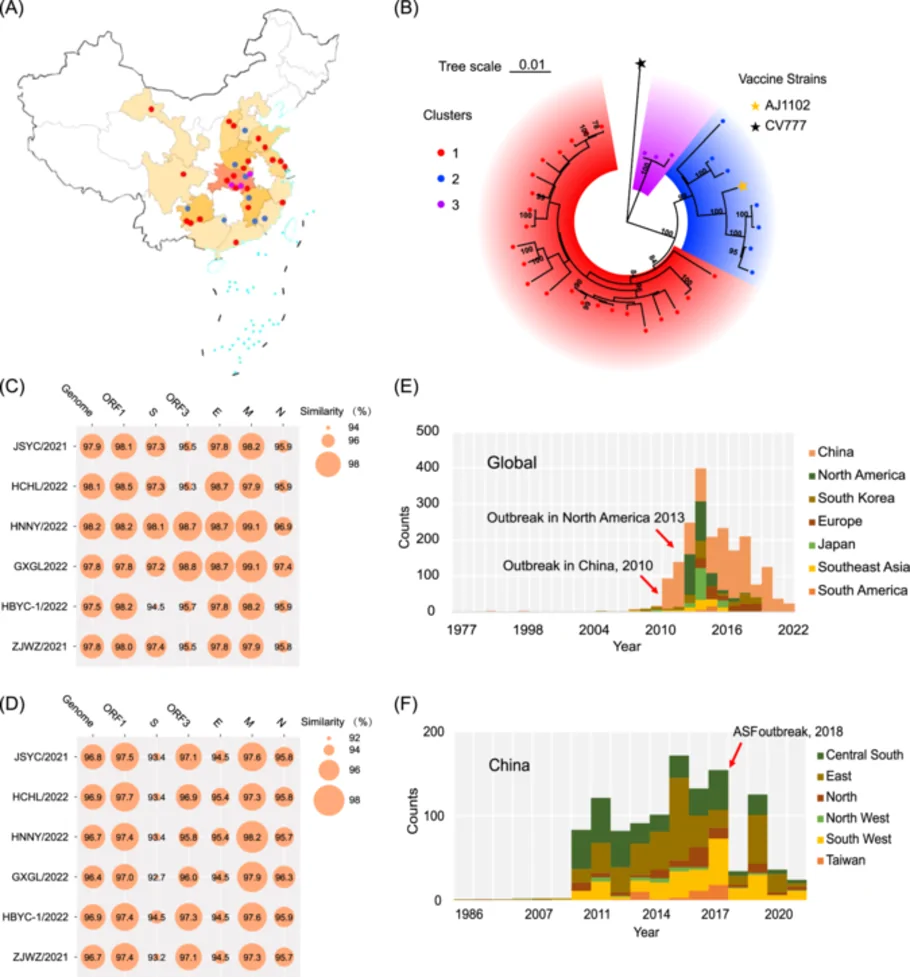
Genetic characteristics of porcine epidemic diarrhea virus (PEDV) in China from 2021 to 2022. (A) Distribution of 34 PEDV strains in this study. Sampling locations are marked by colors that correspond to the respective clusters. The map of China is based on the standard map downloaded from the Standard Map Service Website of the National Natural Resources Department of China http://bzdt.ch.mnr.gov.cn/, with the review number GS(2016)1553. The boundaries of the base map have not been modified. (B) An unrooted phylogenetic tree of 34 S genes with a branch length scale of 0.01. Clusters are highlighted in red, blue, and purple. The vaccine strains are marked by stars. (C, D) Genetic homology of the complete genome, polyprotein ORF1, four structural proteins (S, E, M, and N), and one accessory protein (ORF3) was analyzed between six field strains and two vaccine strains: (C) CV777 and (D) AJ1102‐R. Reporting curves of PEDV S gene sequences globally from 1977 to 2022 (E), and in China from 1986 to 2022 (F).

From 2021 to 2022, we acquired a total of 34 S gene sequences from 14 provinces across the Chinese mainland (Figure 1A), showing nucleotide similarities ranging from 94.0% to 100%. Infomation on the 34 strains is provided in Table S1. In the phylogenetic tree, these strains formed at least three distinct branches, with each branch supported by high bootstrap values exceeding 84% (Figure 1B). The full‐length genomes of six field strains exhibited genetic similarities ranging from 97.3% to 99.8% among themselves. They also showed 97.5% to 98.2% similarities with the vaccine strain AJ1102‐R and 96.4% to 96.9% with the CV777 strain (Figure 1C,D).

From 1977 to 2022, the top four regions reporting PEDV S gene sequences were China (*n* = 1180), North America (*n* = 280), South Korea (*n* = 181), and Europe (*n* = 143). The report curve showed two prominent peaks, corresponding to the outbreaks in China in 2010 and in North America in 2013 (Figure 1E). From 1986 to 2022, the highest number of sequences reported in China originated from the eastern region (*n* = 420), central‐southern region (*n* = 329), and southwestern region (*n* = 243) (Figure 1F). Notably, there was a significant 77.6% reduction in reported sequences from 2018 to 2019, which is likely attributed to the epidemic of African swine fever (ASF) in China in late 2018 (Figure 1F).

### Site polymorphism patterns on PEDV spike protein

**FIGURE 2:**
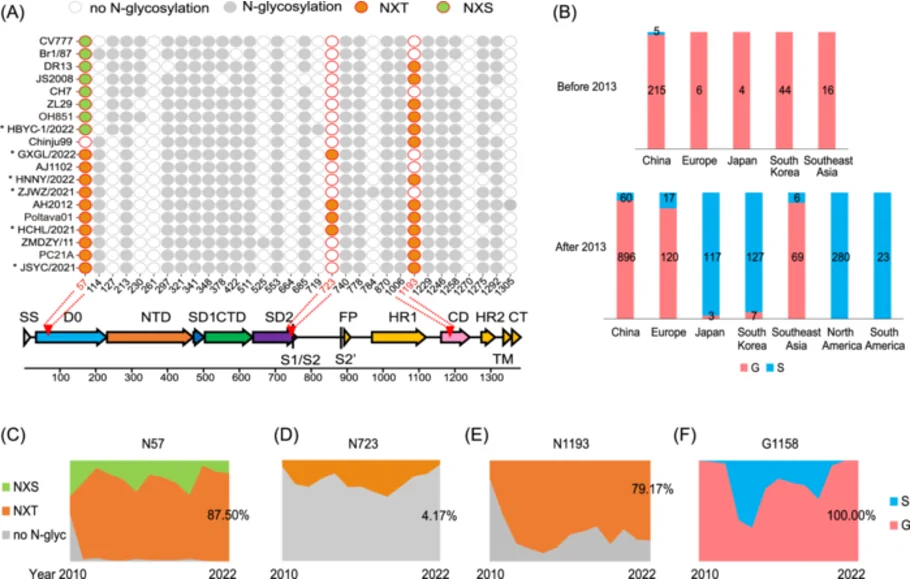
Site polymorphism patterns of the S glycoprotein. (A) Bubble plots depict the distribution of N‐glycosylation sites on the S protein, with putative N‐glycosylation sites denoted with filled bubbles. Three N‐glycosylation sites: N57, N723, and N1193, are specifically indicated on the S protein. The structural domains are labeled as follows: SS (signal peptide), D0 (domain 0), NTD (N‐terminal domain), SD1 (subdomain 1), CTD (C‐terminal domain), SD2 (subdomain 2), HR (heptad repeat), FP (fusion peptide), CD (connection domain), TM (transmembrane region), CT (cytoplasmic tail), S1/S2 (cleavage site of S1/S2), and S' (Furin cleavage site on S2). (B) Distribution of S1158 among PEDV population in different regions before and after 2013. (C−F) Temporal dynamics of the proportions of N57, N723, N1193, and G1158 within the PEDV population from 2010 to 2022.

To further investigate the determinant sites of PEDV genotyping, we primarily selected 13 reference strains with distinct backgrounds and 6 strains from this study to predict N‐glycosylation sites on the S protein. A total of 21 to 24 putative N‐glycosylation sites were identified on the S protein across the 19 PEDV strains examined. Notably, N‐glycosylation at positions 57, 723, and 1193 (N57, N723, and N1193) exhibited distinct polymorphism patterns (Figure 2A). N57 in the D0 domain exhibits two primary forms, NXS and NXT (where X presents any amino acid except proline or asparagine) (Figure S1A). N723 in the SD2 domain, located near the S1/S2 cleavage site, requires the presence of two consecutive NXT motifs from position 719 to 725. Furthermore, a unique NSS at position 720 was identified in the HBYC‐1/2022 strain (GenBank accession no. OQ122100) in this study (Figure S1B). N1193 is situated within the Connection Domain (CD) of the S2 subunit. Mutations or deletions at this position may lead to the elimination of N‐glycosylation. Similar to some early strains observed in Thailand, HNNY/2022 (GenBank accession no. OQ122088) and GZBJ/2021 (GenBank accession no. OQ122113) in this study have acquired an N‐glycosylation at position 1195, thereby replacing the N1193 (Figure S1C). Additionally, a non‐glycosylated site at position 1158 exhibited a distinct geographical trait. Over 98% of the strain population from North America, South Korea, and Japan after 2013 share the substitution G1158S (Figure 2B).

Furthermore, we analyzed the prevalence of these three N‐glycosylation sites and the G1158S substitution among global strains from 2010 to 2022. From 2010 to 2012, strains carrying N57 and N1193 rapidly increased and remained predominant since then, accounting for 87.5% and 79.2% of the strain population in 2022, respectively. In contrast, N723 comprised only a small proportion, representing only 4.17% in 2022. The prevalence of S1158 peaked in 2013 and 2019 but abruptly disappeared in 2022 (Figure 2C−F).

### Genotyping system based on site polymorphism

**FIGURE 3:**
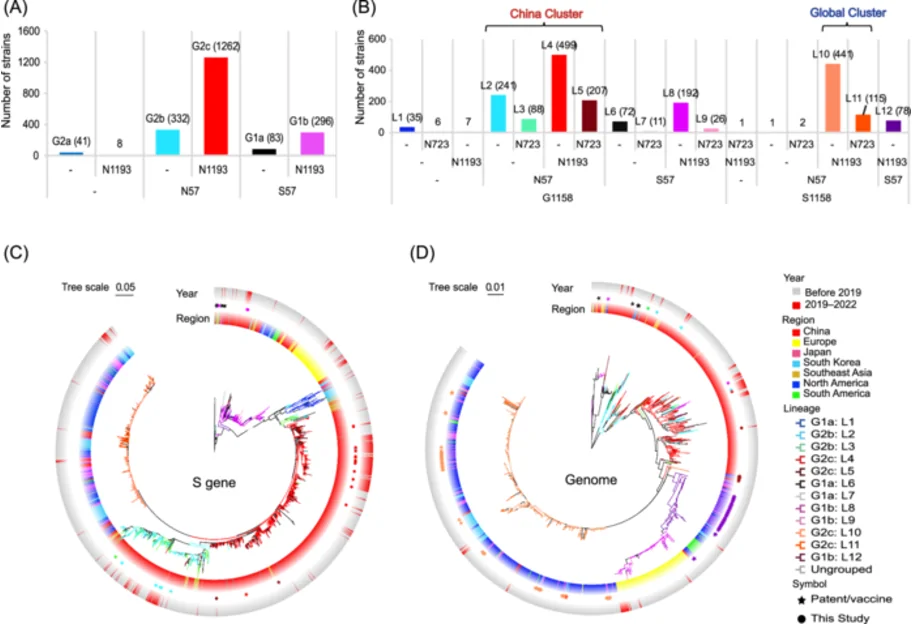
Proposed genotyping system based on site‐specific polymorphism. (A) the N57−N1193 genotyping system and (B) the N57−N1193−N723−G1158 genotyping system. The number of sequences in each genogroup or lineage is presented. Phylogenetic trees based on the genetic sequences of (C) the S genes (*n* = 2022) and (D) the full‐length genomes (*n* = 849) were constructed based on maximum likelihood (ML) method. Lineages are represented by branch color categories. The collection year and countries of origin are indicated by outer color blocks adjacent to the trees. Newly sequenced strains and patent/vaccine strains are denoted with filled circles and stars, respectively.

Next, we attempted to use the mentioned sites to classify PEDV strains. Initially based on N57 and N1193, 2022 PEDV strains could be hierarchically classified into five main genogroups: G1a, G1b, G2a, G2b, and G2c (Figure 3A). Eight strains were assigned to an undefined group owing to the limited sample size. The prevalence of G2c strains was 62.4% (1262/2022), G2b strains accounted for 16.4% (332/2022), and G1b strains constituted 14.6% (296/2022). The proportions of G1a and G2a strains were each less than 5%. Following the incorporation of N723 and G1158 into the classification framework, five genogroups were further subdivided into 12 lineages, L1–L12 (Figure 3B).

On the phylogenetic trees, 97.6% of the L4 and L5 strains, along with 80.9% of the L2 and L3 strains, clustered within a distinct “China Cluster,” while L10 and L11 exhibited over 98% similarity in the S gene with L4 and L5, forming a “Global Cluster” that is predominantly found in North America, South Korea, and Japan. Additionally, L8, L9, and L12 were characterized as less virulent strains and formed clusters that is widespread in Europe, Asia, and the Americas (Figure 3C,D). The 34 strains sequenced in this study in China from 2020 to 2021 were categorized into at least five lineages: L2 (8 strains), L3 (1 strain), L4 (20 strains), L5 (2 strains), and L8 (3 strains).

### Adaptive evolution analysis of PEDV s gene

**FIGURE 4:**
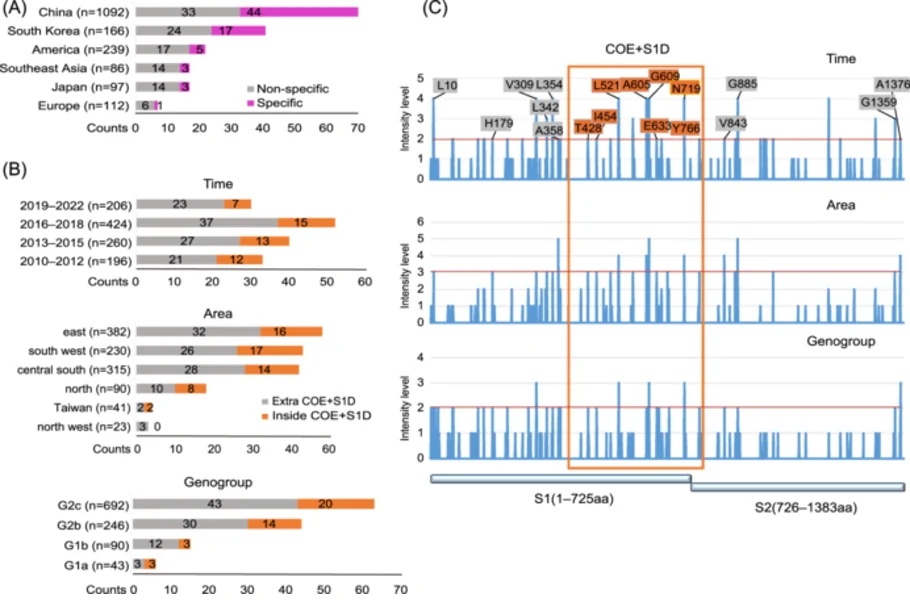
Adaptive selective pressure on the PEDV S protein. (A) Numbers of positive selection sites in different geographic regions. Sites uniquely detected in each region are highlighted in purple. (B) Positive selection sites identified across different time periods, geographic regions, and genogroups in China. (C) Distribution of sites that were under intensity selection. *X*‐axis indicates the location of amino acid sites on the S protein, while the *Y*‐axis indicates the intensity of selection. Amino acid sites that were simultaneously under positive selection from datasets of at least two time periods, three geographic regions, and two genogroups were labeled. The domain of neutralizing epitope, named COE + S1D, was highlighted by an orange box. PEDV, porcine epidemic diarrhea virus.

To further evaluate the selection pressure acting on each amino acid site of the PEDV S protein, bioinformatics methods were employed to analyze the aligned genetic sequences. A total of 77 positive selection sites were identified in the data set of China, including 41 unique sites. Meanwhile, 42 and 27 positively selected sites were detected in the datasets of South Korea and America, respectively (Figure 4A). We categorized the Chinese strains into distinct datasets based on time, region, and genotype, subsequently analyzing the selection pressures within these datasets. The most significant positive selection sites were identified in the temporal data set “2016−2018,” the regional data set “East,” and the genotype data set “G2c,” with 52, 48, and 63 positive selection sites detected, respectively (Figure 4B). We have also identified 18 sites that were under high‐intensity selection (HIS). Eight HIS sites (T428, I454, L521, A605, G609, E633, N719, and Y766) were located within the neutralization epitope region named COE + S1D (amino acid 399 to 789). The frequency of HIS sites within the COE + S1D region was notably higher than in adjacent regions. The density of HIS sites was 2.05 per 100 amino acids in this region, compared to 1.01 sites per 100 amino acids in surrounding regions (Figure 4C). N57 and N1193 were not identified as HIS sites. However, the formation of N‐glycosylation at N723 was strong correlated with the presence of the HIS site at N719 (Figure 4C and Figure S1B). Y766 is situated within the validated neutralizing epitope SS6 [31], where two common substitutions have been observed: Y766S (95.4%) and Y766D (4.0%). Notably, a novel substitution, Y766F, was first identified in the ZJWZ/2021 isolate (GenBank accession no. OQ122101) in this study.

### Discrete phylogenetic analysis that reveals the recombination patterns on PEDV S gene

**FIGURE 5:**
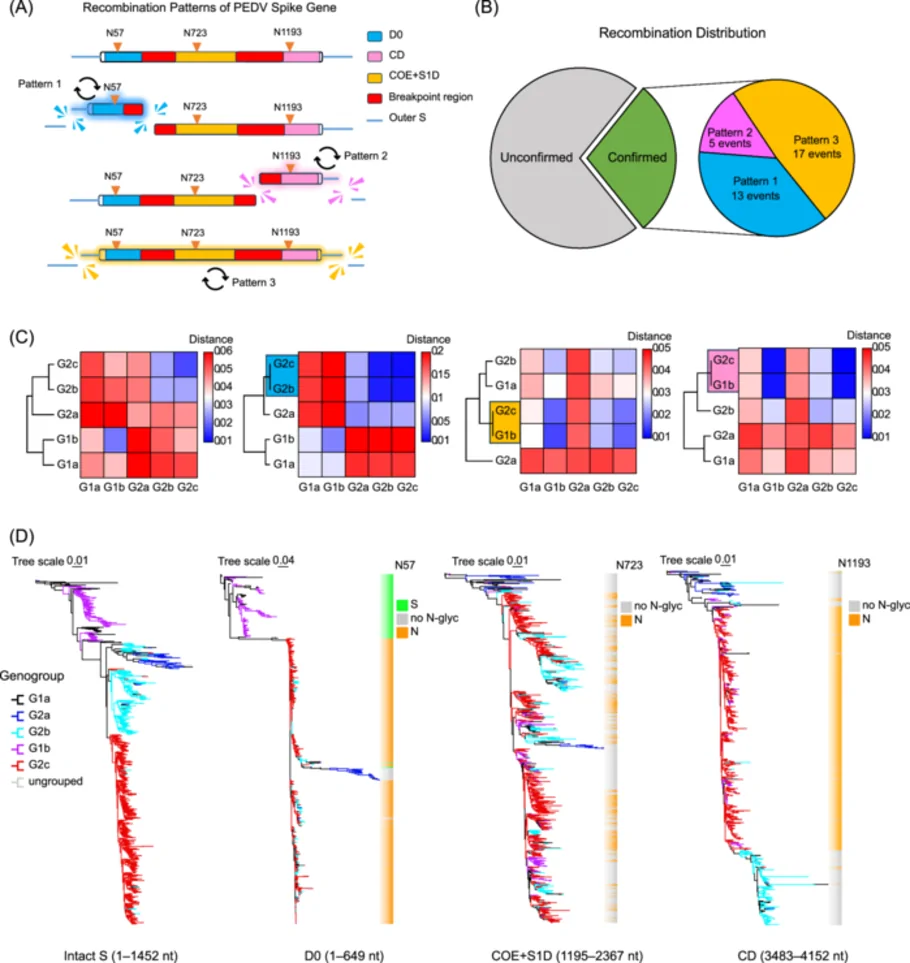
Recombination patterns of the porcine epidemic diarrhea virus (PEDV) S gene. (A) Proposed recombination patterns of PEDV genome. The hotspots of breakpoint on the S genes are indicated in red, while the transferred gene fragments are highlighted. (B) Distribution and frequency of four types of recombination events. (C) Heatmap visualization illustrated the genetic distances among genogroups based on the intact S gene, as well as the D0, COE + S1D, and CD domains. (D) Discrete phylogenetic trees based on the intact S gene, D0, COE + S1D, and CD domains. The branches are color‐coded according to the genogroups. The distributions of N57, N723, and N1193 were represented by color blocks adjacent to the phylogenetic trees.

In addition to investigating adaptive evolution, we also analyzed patterns of recombination in the PEDV genomes. A total of 129 recombination events were detected among 850 PEDV genomes by RDP5, with 35 events having confirmed beginning and ending breakpoints (Table S2). The breakpoints were mainly observed in the downstream nearside of N57 and upstream nearside of N1193, while they were rarely observed in the COE + S1D region (Figure 5A). Since N57 and N1193 were not primarily involved in adaptive evolution, it is hypothesized that these sites may have undergone transfer with gene fragments through recombination events. Consequently, the 35 recombination events can be classified into three distinct patterns: (1) N57 involved, (2) N1193 involved, and (3) the outer S region involved, comprising 13, 5, and 17 events, respectively (Figure 5B).

By deleting the regions of breakpoints, S gene can be segmented into three parts: D0 (1–694 nt), COE + S1D (1195–2367 nt), and CD (3483–4152 nt). The computed mean distances between genogroups revealed that G2c is most closely related to G2b in the D0 domain, with a mean distance of 98.1%. In contrast, G2c exhibits the closest relationship with G1c in both the COE + S1D and CD domains, with a mean distance of 98.2% and 98.9%, respectively (Figure 5C). Subsequently, a discrete phylogenetic analysis was performed using these three domains. Based on the topological structure of the phylogenetic trees, G2c was found to cluster with G2b in the D0 region, whereas it clustered with G1b in the CD regions. However, genogroups intersect with each other without forming distinct clusters in the COE + S1D region (Figure 5D). The distribution patterns of N57 and N1193 were in accordance with the clustering of genotypes (Figure 5D). Furthermore, we observed 45 sites associated with N57 and eight sites with N1193. These glycosylation‐associated sites exhibited co‐evolving substitution patterns with the polymorphisms of N57 and N1193 (Table S3).

### Reconstruction of the global epidemic dynamics of PEDV lineages

**FIGURE 6:**
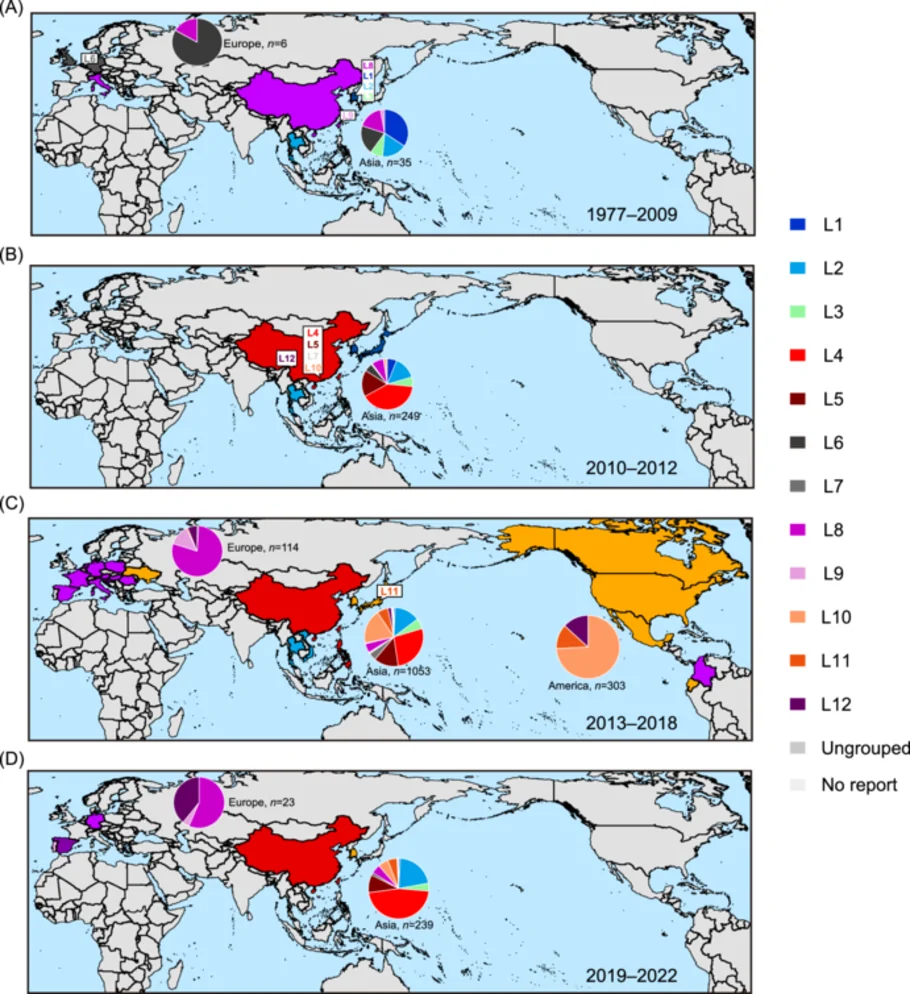
The epidemic map of global porcine epidemic diarrhea virus (PEDV) across different time periods: (A) 1977−2009, characterized by origins in Europe and early dissemination in Europe and Asia; (B) 2010−2012, marked by outbreak of virulent strains in Asia; (C) 2013−2018, designated as the “Global Epidemic” period; and (D) 2019−2022, reflecting the current prevalence status of PEDV. Blank areas on the map indicate the absence of reported PEDV S gene sequences in this region. Countries were color‐coded according to the predominant PEDV S gene lineage reported. The pie chart illustrates the temporal distribution of reported S gene sequences and the proportions of different lineages across Asia, Europe, and the Americas over time. The initial reporting location of each lineage was indicated on the map.

The epidemic history and evolutionary dynamics of PEDV were reconstructed using spatiotemporal data of global strains. The first reported strains from each lineage have been listed (Table S4). The classical lineage L6 first emerged in Europe in the 1970s and subsequently spread to various Asia countries. Over the two‐decade period from 1998 to 2009, several novel lineages, including L1, L2, L3, and L8, were identified (Figure 6A). L1 and L8, which were initially identified in South Korea in 1998, exhibited 94.2% and 99.7% similarity with classical strains in the S gene, respectively. Additionally, it was observed that L2 and L3 had been circulating in Thailand before 2010 (Figure 6A). Between 2010 and 2012, at least five new lineages—L4, L5, L7, L10, and L12—emerged in China (Figure 6B). Notably, L4, which likely originated through recombination, rapidly became the predominant lineage in the Chinese mainland since its emergence, comprising 43.3% (482/1112) of the prevalent strain in the Chinese mainland. From 2013 to 2018, PEDV emerged and re‐emerged in multiple countries across the America, Europe, and Asia, marking this period as the “Global Epidemic” period (Figure 6C). During this time, the L10 was predominant among the prevalent strains in the United States (80.6%, 175/217), Japan (74.2%, 89/120), South Korea (66.4%, 79/119), and Mexico (64.9%, 37/57). In contrast, apart from a single report of the L11 in Ukraine, the less virulence L8 lineage was predominant in Europe, comprising 78.9% (90/114) of the strain population. From 2019 to 2022, the number of countries reporting PEDV and the sequences both declined. However, in the Asian region, especially in China, the diversity of PEDV strains remained at a relatively high level (Figure 6D).

## 3 DISCUSSION

PED has been characterized as “emerging” and “re‐emerging” disease on a global scale [32]. In “emerging” countries like the United States and Canada, which had pig population that were naive and highly susceptible to PEDV, the virus spread rapidly upon introduction, leading to significant losses. In “re‐emerging” countries such as China and South Korea, variant PEDV strains have the potential to break through the established herd immunity or biosecurity measures, resulting in recurrent outbreaks. Here, we conducted a 2‐year investigation of PEDV in China and found high genetic diversity among the prevailing strains (Figure 1A−C), which aligns with finding from a previous study [33]. Moreover, this high level of diversity has persisted since the outbreak in China in 2010 [11], posing a significant challenge to the prevention and control of PED. Understanding the epidemic dynamics and viral evolution of PED holds significant importance for accurate diagnosis and effective immunity strategies.

Currently, the widely accepted classification methods of PEDV are based on the branches of phylogenetic trees [16, 34]. However, genotyping based on phylogeny presents several challenges as the outcomes are sensitive to the number of sequences analyzed. Furthermore, the accuracy of topological tests of the clades can be influenced by both evolutionary histories and recombination of the genes [35]. In another study, PEDV strains were classified into “non‐S‐INDEL” and “non‐S‐INDEL” based on specific insertions and deletions at the N‐terminus region of the S1 gene [36]. Considering the critical role of the S2 subunit of coronaviruses in both functionality and antigenicity [37, 38], we conclude that the intact S gene is indispensable for accurate genotyping of PEDV. Herein, we proposed an innovative system that leverages the site polymorphism patterns across intact S genes, demonstrating stability and accuracy in the classification of PEDV strains. Over 2000 historical global strains of PEDV were classified into five genogroups (G1a, G1b, G2a, G2b, and G2c), which align with the branches on the phylogenetic tree (Figures 3C and Figure 5D). Furthermore, 12 PEDV lineages that vary in geographic distribution have been identified. To better classify strains with both genotype and lineage, a taxonomy denoted as Gx: Lx (Genogroup x: Lineage x) was introduced (Figure 3D).

The spike protein of coronaviruses has extensive N‐glycosylation sites on its surface that are involved in conformational regulation and immune evasion [19, 20]. A previous study has confirmed the presence of N‐glycans at positions N57, N723, and N1193 on the PEDV S protein [39]. The N‐glycans of N57, situated at the top outside of the S protein, may play a crucial role in modulating the “up” and “down” conformation of the D0 domain [39, 40]. The N‐glycan of N1193, situated on the S2 subunit, connects the protein fusion regions HR1 and HR2. Although the specific biological function of the CD domain has not been fully elucidated, studies on other coronaviruses suggest that the homeodomain plays a role in anchoring the fusion machinery during cell fusion process [20, 41]. Therefore, mutations at N1193 may have a potential influence on the stability of the fusion machinery. Interestingly, we have identified 45 sites at the N‐terminus along with eight sites at the C‐terminus of the S protein that co‐vary with N57 and N1193, named glycosylation‐associated sites, which include two additional N‐glycosylation sites: N114 and N127 (Table S3). Although we hypothesize that sites N57 and N1193 are strongly associated with the viral phenotype, validating this association with only N57 or N1193 may not be sufficient due to their joint effects with these co‐vary sites. A notable feature of the S glycoprotein is the reduced glycosylation observed around the proteolytic cleavage sites S1/S2 and S2'. This reduction likely facilitates efficient protease cleavage and subsequent membrane fusion [18]. Therefore, the N‐glycosylation at N723, which is proximal to the S1/S2 cleavage site, may modulate the accessibility and activation of host proteases. Additionally, given its location within the neutralizing epitope region, N723 may play a potential role in viral immune evasion [42]. Strains responsible for outbreaks in the United States, Canada, South Korea, and Japan after 2013 likely share a common ancestor due to their high genetic identity [43, 44, 45]; they all acquired the G1158S substitution (Figure 2B). Notably, the initial strain with G1158S substitution was GDFS/2011 (GenBank accession no. KF546800) reported in Guangdong, China, in 2011, 2 years before the identification of a highly similar strain Colorado/2013 (GenBank accession no. KF272920) during the outbreak in the United States. The genetic similarity between these two strains exceeded 99.5%. The evidence of timeline and homology suggests a potential dissemination route of PEDV from China to the United States (Figure 6B,C). However, strains with G1158S substitution, such as lineage L10, have not become predominant in the Chinese mainland, indicating that stochastic events might play a significant role in the epidemics of PEDV in specific regions [28]. Overall, site polymorphism may serve as a robust molecular marker, offering a more comprehensive explanation of epidemic dynamics and genetic divergence of PEDV.

Adaptive evolution of the S protein may alter the pathogenicity and antigenicity of coronaviruses [46, 47]. Selective analysis is typically employed to infer adaptive evolution in response to pressure from host immunity or ecology factors [48]. In this study, two computational methods grounded in phylogenetic analysis were employed to estimate the intensity of natural selection acting on PEDV S protein. Despite the removal of duplicate samples as a pre‐processing step for the analysis, the extended sampling period and the expansive sequences datasets may have contributed to an increased number of detectable positive selection sites, as observed in the Chinese data set (*n* = 1092) (Figure 4A,B). Fewer positive sites were detected in the United States data set (*n* = 239) compared to the South Korean data set (*n* = 166). This difference may be attributed to the reporting of numerous similar sequences in the United States within a short period from 2013 to 2015. However, the fewest positive selection sites were observed among the 112 European samples collected over a long period, suggesting that adaptive evolution may differ between regions (Figure 4A). A total of 18 HIS sites were identified. We observed that the HIS sites were predominantly localized within or near the central region of the S protein, including the COE + S1D domain (Figure 5C). This region is regarded as a hotspot for adaptive evolution as it potentially encompasses the receptor‐binding domain of PEDV [49], which interacts frequently with both host receptors and immune system. In contrast, N57 and N1193, along with most of the glycosylation‐associated sites located at both termini of the S protein, were unlikely to undergo adaptive selection, except for sites V309, G1359, and V1376 (Figure 5C and Table S3).

Recombination represents an important evolutionary pathway of coronaviruses evolution, potentially facilitated by the template‐switching activity of RNA‐dependent RNA polymerase (RdRp) during RNA transcription [50, 51]. Various recombination events have been detected among different PEDV strains using RDP software [22]. In addition to bioinformatic evidence, Cassandra et al. [52] demonstrated recombinant RNAs by experimentally co‐infecting cells with genetically distinct coronaviruses. Song et al. [53] successfully rescued a recombinant PEDV strain, HSGP, by co‐inoculating Vero cells with both a virulent and an attenuated strain. In our study, we observed a sharp shift in the branching patterns on the phylogenetic trees of PEDV (Figure 3B), similar to the reassortment patterns observed in the HA genes of influenza virus and VP7 genes of rotavirus [3, 54]. Furthermore, utilizing RDP4 software, we observed an uneven distribution of breakpoints within the S gene, and two potential breakpoint hotspots were identified. By deleting the breakpoint regions, the S gene was segmented into three distinct fragments. Subsequently, we observed unique recombination patterns within these three fragments using a discrete phylogenetic analysis. The recombination patterns of the S gene can be described as frequent exchange of the terminal regions between various strains (Figure 5A). Although we are currently unable to explain the biological mechanism underlying the recombination patterns, we hypothesize that the secondary and tertiary structure of RNA may serve as a determinant. Further investigation can be conducted utilizing advanced RNA structure prediction tools such as RhoFold+ [55].

The discrete phylogenetic analysis offers compelling evidence suggesting that G2c strains likely share a common ancestor in the D0 region with G2b genogroup and CD region with G1b genogroup (Figure 5C,D). Following its emergence after 2010, G2c most likely originated from the recombination of G1b and G2b genogroups. Additionally, early recombination events may have been subsequently refined through adaptive evolution. For example, the insertion of TGENQ or ΔΔKNQ at position 55 of the N‐terminus of S1 in prevalent strains in 2010 may represent an evolutionary transition to the stable insertion (IGENQ) observed in virulent strains (Figure S1A). The formation of N‐glycosylation N1193 underwent a transition from TYT to NYT and subsequently to NHT. In contrast, N‐glycosylation at position N1195 observed in some strains from Thailand and the newly sequenced strain HNNY/2022 (GenBank accession no. OQ122088) in this study, showing an alternative evolutionary process (Figure S1C). In addition, a recent study has documented the emergence of recombinant strains within the G1b subgroup [56]. Therefore, we proposed that recombination between strains occurs in an undirected manner, allowing fragments of the D0 and CD domains to be transferred in or out between genogroups. This continuous exchange results in the assembly of novel strains with phenotypic characteristics inherited from both parental strains.

Multiple‐strain infections involving distinct strains or the frequent administration of live attenuated vaccines (LAV) may promote recombination among PEDV [57]. In this context, we emphasize using LAV genetically matched to the local strain in swine farms to prevent potential recombination between vaccine and field strains. While whole‐herd feedback can effectively stimulate mucosal immunity in pregnant sows and reduce piglet mortality [58], this measure should be implemented cautiously. It may facilitate the long‐term existence and circulation of PEDV within a farm, accelerating the adaptive evolution of PEDV [59].

We acknowledge that this study has some potential limitations. For instance, while the newly sequenced S genes from 2021 to 2022 provided valuable complementary information to enhance our understanding of PEDV prevalence in China, the relatively limited quantity and timeliness of sequences obtained may have restricted the comprehensiveness and accuracy of the analysis. However, by incorporating a substantial number of global sequences, we were able to reconstruct the evolutionary dynamics of PEDV. While preparing the manuscript, we have continuously collected data from clinic samples and public databases and found that recent strains remain consistent with our genotyping system. Nonetheless, to maintain the integrity and completeness of our existing analysis and conclusions, we chose not to incorporate this additional data into the manuscript. Additionally, our classification based on site polymorphism may not encompass some validated key epitopes [31, 60]. On the other hand, it provides a flexible framework that can be expanded to incorporate additional sites for genotyping. Since our study is a theoretical investigation grounded in bioinformatic analysis, further phenotypic studies are essential to refine and validate this system.

## 4 CONCLUSION

In summary, based on analysis of a large‐scale data set of gene sequence information, our study systematically classified PEDV, reconstructed its global epidemic dynamics, and provided novel insights into the evolutionary pathway of the coronavirus S protein.

Our findings not only provide a robust framework for PEDV classification but also enhance our understanding of coronavirus evolution, which could inform future vaccine development and epidemic control strategies.

## 5 METHODS

### Sample collection and sequencing

From January 2021 to November 2022, a total of 205 samples of intestinal tissues or feces were collected from pigs exhibiting diarrheal symptoms (Figure S2). The S gene of PEDV was amplified by using the forward primer: 5′‐TCTTCTGGCGTAATTCCACAAT‐3′ and the reverse primer: 5′‐GCCTCAAAGAAGACGCTTTAAAC‐3′. The full‐length genome of PEDV was sequenced by conventional PCR with 28 pairs of primers, as previously described [61].

### Comparative genomic and site polymorphism analysis

A total of 1988 PEDV S genes and 843 genomes up to August 15, 2022, were retrieved from the Virus Pathogen Database and Analysis Resources [62]. All information regarding the sequences utilized for analysis, including GenBank accession numbers, taxonomy, collection years, and countries of origin, are provided in Table S5. Nucleotide and deduced amino acid sequences were aligned using MAFFT v7.505 [63]. The genetic distance was calculated using the Maximum Composite Likelihood model in MEGA7 with default parameters [64]. Putative N‐glycosylation sites were predicted using the NetNGlyc‐1.0 server with a threshold of 0.5 [65].

### Phylogenetic analysis

Phylogenetic trees of PEDV genetic sequences were constructed using the maximum likelihood (ML) method with 1000 bootstrap replicates in FastTree v2.1.11 [66]. Branches with bootstrap values higher than 70 were regarded as robustly supported [67]. Sequences with similarities greater than 99.5% were removed using BioAider v1.527 before the discrete phylogenetic analysis [68]. The results of the phylogenetic analysis were visualized using the tvBOT tool on Chiplot (https://www.chiplot.online/) [69].

### Selective analysis

To infer the selective pressure on the S gene, we employed both Mixed Effects Models of Evolution (MEME) to test episodic selection and Fast Unconstrained Bayesian AppRoximation (FUBAR) to test pervasive selection sites [70, 71]. Sites with a *p*‐value ≤ 0.1 in MEME and a posterior probability ≥ 0.9 in FUBAR were considered as being under positive selection. To further identify the sites subjected to high‐intensity adaptive selection, the S gene sequences were categorized into different datasets according to time periods, geographic regions, and genogroups. All Chinese administrative provinces were categorized into five distinct regions according to the Zonal Prevention and Control Policy for ASF and other Significant Swine Disease, implemented by the Ministry of Agriculture and Rural Affairs in April 2021, https://www.gov.cn/zhengce/zhengceku/2021‐04/22/content_5601271.htm. Sequences from Taiwan were categorized as a separate data set. Sites that were positively selected in ≥2 time periods, ≥3 geographic regions, and ≥2 genogroup datasets were defined as high‐intensity selection sites.

### Recombination analysis

Recombination detection program 5 (RDP5) was used to detect recombination events in PEDV [72]. Regions of sequences with poor alignment performance were automatically trimmed using trimAl v1.2 before analysis [73]. Seven methods in RDP5 were employed for the formal recombination tests: RDP, MAXCHI, CHIMAERA, 3SEQ, and GENECONV (as primary methods), along with BootScan and SiScan (as secondary methods). All methods were run with the default settings as recommended. Accepted recombination events should pass five out of the seven methods, with each method achieving a *p*‐value threshold of less than 0.05. We manually checked the distribution of breakpoints within the S gene using 99% confidence intervals. Subsequently, the patterns of genetic fragments exchange were characterized based on the specific locations of these breakpoints.

## AUTHOR CONTRIBUTIONS

**Mingkai Lei**: Writing—original draft; conceptualization; data curation; methodology; software; visualization; investigation; writing—review and editing; formal analysis. **Huimin Li**: Investigation; conceptualization; methodology; data curation. **Xiaoyu Chen**: Validation; writing—review and editing. **Xiaozhen Li**: Resources; funding acquisition. **Xuexiang Yu**: Investigation; data curation; resources. **Shengnan Ruan**: Validation; writing—review and editing. **Hao Wu**: Validation; writing—review and editing. **Ahmed H Ghonaim**: Writing—review and editing; formal analysis. **Ziyang Yan**: Investigation; software. **Wentao Li**: Writing—review and editing; conceptualization; supervision; validation. **Qigai He**: Validation; project administration; supervision; funding acquisition; writing—review and editing; conceptualization.

## CONFLICT OF INTEREST STATEMENT

The authors declare no conflicts of interest.

## ETHICS STATEMENT

No animals or humans were involved in this study.

## Supporting information

Figure S1 Polymorphisms of N57, N723, and N1193.
Figure S2 Clinical symptoms caused by the infection of different PEDV strains.

Table S1 Information of strains sequenced in this study.
Table S2 List of confirmed recombination events.
Table S3 List of N‐glycosylation‐associated sites.
Table S4 Early strains of 12 PEDV lineages.
Table S5 List of global sequences used for building genotyping system.

## Data Availability

The data that supports the findings of this study are available in the supplementary material of this article. All the sequencing data have been deposited in NCBI under GenBank accession numbers OQ122084−OQ122117 (S gene sequences of 34 PEDV strains), PP472642−PP472646 (full‐length genome of 6 PEDV strains). The data can be find in the URL of NCBI collection https://www.ncbi.nlm.nih.gov/sites/myncbi/1Z9QtLM1v9uATH/collections/65122944/public/. Supplementary materials (figures, tables, graphical abstract, slides, videos, Chinese translated version and updated materials) may be found in the online DOI or iMetaOmics Science http://www.imeta.science/imetaomics/.
