# Correction to “A Novel Genotyping System Based on Site Polymorphism on Spike Gene Reveals the Evolutionary Pathway of Porcine Epidemic Diarrhea Virus”

**DOI:** 10.1002/imo2.70049

**Published:** 2025-08-21

Lei, Mingkai, Huimin Li, Xiaoyu Chen, Xiaozhen Li, Xuexiang Yu, Shengnan Ruan, Hao Wu, et al. 2025. “A Novel Genotyping System Based on Site Polymorphism on Spike Gene Reveals the Evolutionary Pathway of Porcine Epidemic Diarrhea Virus.” *iMetaOmics* 2025: e70013. https://doi.org/10.1002/imo2.70013.

Wuhan Keqian Biology Co., Ltd. and the Diagnostic Center for Animal Disease of Huazhong Agricultural University are qualified institutions for third‐party diagnostics. All samples in this study were collected by technicians on pig farms and subsequently sent to these institutions. We obtained permission to use these samples after the diagnostic services were completed.

In the “METHODS” section, the original text “From January 2021 to November 2022, a total of 205 samples of intestinal tissues or feces were collected from pigs exhibiting diarrheal symptoms” was ambiguous and could lead reviewers and readers to assume that the samples were collected by the authors. We would like to correct this sentence to: “From January 2021 to November 2022, a total of 205 samples of intestinal tissues or feces from pigs exhibiting diarrheal symptoms were provided by Wuhan Keqian Biology Co., Ltd. and the Diagnostic Center for Animal Disease of Huazhong Agricultural University.”

Since the sampling was not directly performed by the authors, an ethics statement was oversighted. An ethical application was requested by the editor and the approval was granted after the publication. In this statement, we have also clarified the exact period of the experiment as well as the sources of the samples.

In the “ETHICS STATEMENT” section, the original text “No animals or humans were involved in this study.” was not appropriate and should be revised to: “The ethics application (No. HZAUSW‐2025‐0065) was approved by the Ethics Committee of Huazhong Agricultural University.”

We apologize for this error.

