# Supplementary material for: A novel genotyping system based on site polymorphism on spike gene reveals the evolutionary pathway of porcine epidemic diarrhea virus
Source: IMetaOmics. 2025 Apr 6;2(2):e70013. doi: 10.1002/imo2.70013 (PMC12806362; doi:10.1002/imo2.70013)
Supplement: Supplementary file 1 — Figure S1 Polymorphisms of N57, N723, and N1193. Figure S2 Clinical symptoms caused by the infection of different PEDV strains. [file IMO2-2-e70013-s001.docx]

Supporting Information to

**A novel genotyping system based on site polymorphism on spike gene reveals the evolutionary pathway of porcine epidemic diarrhea virus**

**Running title:** Spike Gene Polymorphism Reveals PEDV Evolution

Mingkai Lei^1^, Huimin Li^1^, Xiaoyu Chen^1^, Xiaozhen Li^2^, Xuexiang Yu^1^, Shengnan Ruan^1^, Hao Wu^1^, Ahmed H Ghonaim^1,3^, Ziyang Yan^1^, Wentao Li^1,4^*, Qigai He^1^*

^1^National Key Laboratory of Agricultural Microbiology, College of Veterinary Medicine, Huazhong Agricultural University, Wuhan 430070, China

^2^Yunnan Southwest Agricultural and Animal Husbandry Group Co, Ltd, Kunming 650224, China

^3^Desert Research Center, Cairo 11435, Egypt

^4^Hubei Hongshan Laboratory, Wuhan 430070, China

***Correspondence:** [he628@mail.hzau.edu.cn](mailto:he628@mail.hzau.edu.cn) (Qigai He); [wentao@mail.hzau.edu.cn](mailto:wentao@mail.hzau.edu.cn) (Wentao Li)


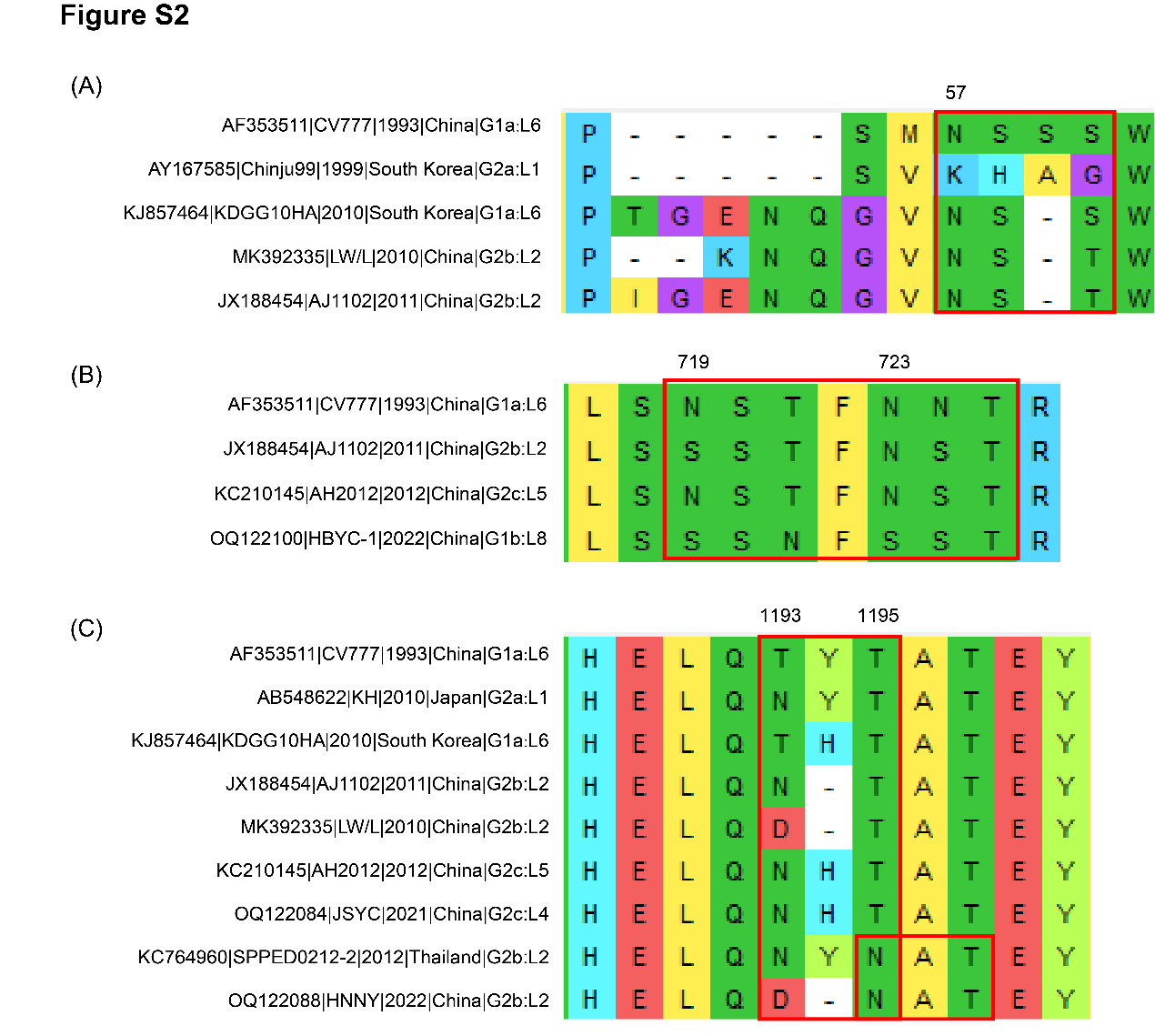


**Figure S1 Polymorphisms of N57, N723 and N1193.** Demonstration of amino acid sequence polymorphism at positions (A) 57, (B) 723, and (C) 1193. Red boxes refer to the N-glycosylation sequences. Reference strains with information of accession number, strain name, year of collection, group, and lineage are on the left.


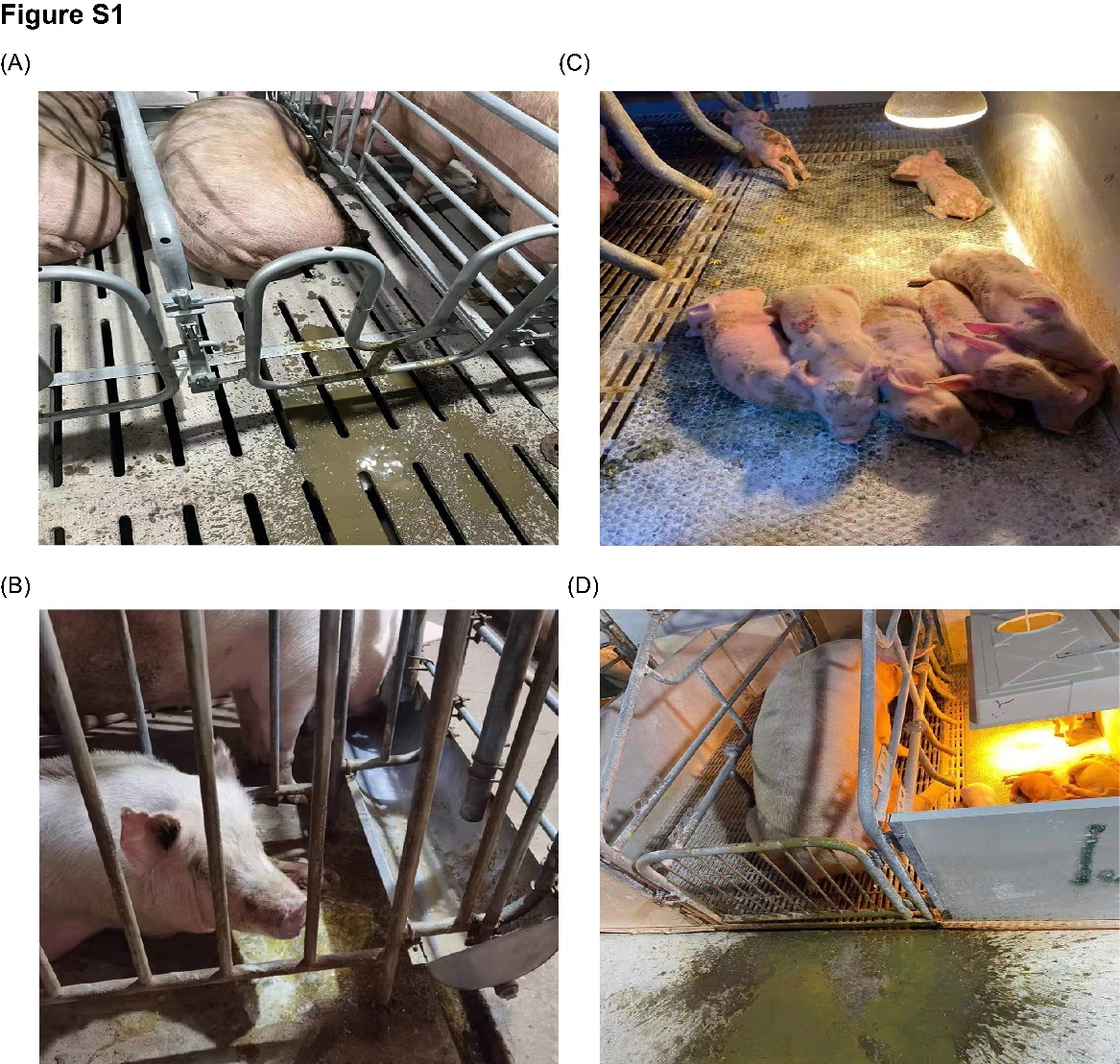


**Figure S2 Clinical symptoms caused by the infection of different PEDV strains.** (A,B) G1b: L8 strain HBYC-1/2022 causes vomiting and watery diarrhea in sows. (C,D) G2c: L4 strain JSYC/2021 causes diarrhea and dehydration in sows and suckling piglets in the farrowing house.
